# Treats containing cannabidiol, L-tryptophan and *α*-casozepine have a mild stress-reducing effect in dogs

**DOI:** 10.3389/fvets.2025.1632868

**Published:** 2025-07-29

**Authors:** Hannah E. Flint, Jennifer E. Weller, Alysia B. G. Hunt, Tammie King

**Affiliations:** Waltham Petcare Science Institute, Melton Mowbray, United Kingdom

**Keywords:** cannabidiol, CBD, tryptophan, *α*-casozepine, dog, stress, transportation, animal welfare

## Abstract

**Introduction:**

Demand for stress-reducing products aimed at pets has risen in recent years, demonstrated by an ever-growing market of nutritional and odor-based products. Previous research has demonstrated an effect of Cannabidiol (CBD), L-tryptophan and *α*-casozepine on stress-related behaviours in a variety of animal species, including dogs. The objective of this study was to explore the efficacy of a treat product containing two different doses of CBD (2 mg/kg BW and 4 mg/kg BW) in addition to a 2mg/kg BW dose of CBD combined with L-Tryptophan and *α*-casozepine (blend) in comparison to a placebo.

**Methods:**

A blinded cross-over study was performed in which 54 dogs received a single dose of each treatment two hours prior to exposure to a previously developed stress paradigm (10 min. car travel). A range of behavioural and physiological measures were collected pre/post (plasma CBD levels, serum cortisol) or during (heart rate, heart rate variability, surface temperature, activity, posture, stress-related behaviours, qualitative ratings) the stress paradigm.

**Results:**

All treatments resulted in elevated post-test CBD levels in the plasma in comparison to placebo (*p <* 0.001), the 4 mg/kg BW CBD had higher post-test CBD levels in comparison to the 2 mg/kg BW CBD without the blend (*p* = 0.002). Furthermore, the 2 mg/kg BW CBD combined with the blend treatment resulted in a significantly smaller increase in cortisol from baseline to post-stress (*p* = 0.016) in response to car travel in comparison to the placebo. However, no other significant effects of treatment were observed, and CBD plasma levels were highly variable between individual dogs, which may have impacted results.

**Discussion:**

CBD combined with the blend had a mild stress-reducing effect in dogs. Further exploration of the efficacy of CBD in reducing stress and anxiety, including interactions with different active ingredients and individual differences in absorption and metabolism are warranted.

## Introduction

Pet dogs are likely to experience a wide range of stressful situations over the course of their lives, which can be exacerbated by the increasing expectation of dogs to fit into humans’ lifestyles. Stress is commonly defined as an organism’s response to, and efforts to cope with, an internal or external threat (1). For example, many dogs are frequently required to travel by car to accompany their owners on vacations, visit nearby walking areas, or attend veterinary treatments (2). A study by Mariti et al. (1) suggested that one in four dogs suffer from travel-related problems when transported via a car, potentially due to high temperatures, loud noises, and intense vibrations (3). In addition, being restrained within a vehicle restricts a dog’s freedom to control their physical and social environment, which can further increase stress.

Previous research has demonstrated that blood serum cortisol, heart rate and the frequency of whining, lip-licking, and yawning were elevated during car travel when compared to baseline measures, suggesting automotive transport is likely to be stressful for some dogs (4, 5).

Stress, in both people and their pets, is rapidly becoming a common cause for concern, with the consumer market showing an uptake in support for products that claim to improve pet emotional wellbeing based on calming or stress-reducing effects (6–8). In addition to on-going research being conducted in humans, recent animal-focused research has highlighted the potential of Cannabidiol (commonly referred to as CBD) as a solution for managing stress and anxiety in pets (7, 9, 10). CBD is one of many non-psychoactive cannabinoids derived from the multi-purpose hemp plant (*Cannabis sativa* L.) that activates the endocannabinoid system of both humans and non-human animals (11, 12). Although the full pharmacological mechanism of CBD has yet to be determined (10, 13), previous research has demonstrated its effectiveness in managing numerous physical and mental health conditions in people including pain, sleep, anxiety disorders, and neurogenerative disorders (13–17).

In addition, CBD has been shown to be safe for use in healthy adult dogs, with single oral doses of 10 to 62 mg/kg body weight (BW) (18, 19) and long-term daily oral doses of 4 mg/kg BW for up to 6 months (20). When exploring the efficacy of CBD in dogs, most studies have focused on physical health benefits, such as reduced pain, increased activity, and improved quality of life in osteoarthritic individuals (21–24). However, only a limited number of studies have explored the efficacy of CBD as an anxiolytic in dogs and the results have been mixed (4, 5, 25, 26). Morris et al. (26) observed no difference in dogs blood cortisol levels after exposure to a simulated firework paradigm when provided with a 1.4 mg/kg BW dose. This is likely due to the administration of CBD too prematurely (i.e., ~4–6 h prior to testing), as multiple studies have reported peak CBD levels in the blood between 1.5 and 2 h after dosing (18, 22, 27). Another study treated shelter dogs with 0.5 drops of CBD oil per kg BW over 45 days and observed a significant reduction in aggressive behavior over time, however this decrease was not significantly different from the placebo group, and no change in stress-related behaviors were observed (25). However, a combined six-month safety and efficacy study in dogs concluded that the provision of daily oral doses of 4 mg/kg BW were safe and efficacious in reducing stress for healthy adult dogs (4, 5, 20). Specifically, administration of a single 4 mg/kg BW dose of CBD resulted in dogs being scored as significantly less “sad” using qualitative behavior assessments during both car travel and separation tests (5). Dogs also spent less time sitting and whining, and traveled a further distance during the separation test, after their first dose of CBD. Finally, dogs were found to have a smaller increase in blood serum cortisol after having experienced car travel when given their first dose of CBD compared to the placebo group (5), suggesting a reduction in stress. When the efficacy of repeated CBD dosing over 24 weeks was investigated while continuing to expose dogs to car travel, results revealed that while the provision of CBD continued to have a mild anxiolytic effect on dogs, repeated exposure to car travel resulted in a decreased stress response from dogs given the placebo (4). This habituation resulted in decreased treatment effects being observed at the final timepoint.

In addition to CBD, there are other compounds that have the potential to improve emotional wellbeing in pets. A range of odor-based products are readily available for reducing stress in both people and pets, including valerian, lavender, and chamomile, as well as synthetic pheromones, such as ‘dog appeasing pheromone’ (DAP). However, more scientific evidence is required to support the efficacy of these ingredients in dogs (reviewed by (28)). Two active ingredients (L-tryptophan and *α*-casozepine), which have previously been shown to demonstrate anxiolytic effects in a variety of species (29–34), were identified as potential candidates for further research which could be used in combination with CBD to alleviate stress in pet dogs.

*Α*-casozepine is a milk protein that originates from the *α*-S1 casein portion of milk and is also referred to by the registered name Lactium® when in its manufactured form. This protein has a structure homologous to benzodiazepines and is thought to have a mechanism of action through binding of the GABA_A_ receptor (35). In a blinded randomized multi-center study comparing the effect of both *α*-casozepine (15 mg/kg BW) and selegiline (0.5 mg/kg BW; a product marketed in veterinary medicine for the treatment of anxiety disorders and age-related cognitive changes), a similar improvement in owner reported emotional disorder evaluation in dog (EDED) scores were observed for both treatments (30). Additionally, a blinded study conducted on laboratory beagle dogs demonstrated that anxious dogs fed a diet containing *α*-casozepine (dosage not reported) exhibited an improvement in behavioral and cortisol responses to the presence of the investigator and an open field test after 65 days of treatment (36).

L-tryptophan is an essential amino acid precursor of serotonin. The serotonergic system plays an important role in regulating mood and behavior (37), and decreased levels of serotonin have been linked to aggressive behavior in dogs (38, 39). Furthermore, fearful dogs have been demonstrated to have altered tryptophan metabolism in comparison to non-fearful controls (40). However, studies looking at supplementing diets with L-tryptophan have had mixed results. One study found significant improvement in aggressive behaviors in dogs following feeding of diets supplemented with L-tryptophan [1.45 g/kg of diet; (41)]. On the other hand, studies looking at the effect of supplementary L-tryptophan on anxiety (42) and obsessive-compulsive behaviors (43) found no significant effects.

L-tryptophan and *α*-casozepine combined within a dry diet (Royal Canin CALM CANINE) have previously been tested in privately owned dogs observed to display signs of anxiety during a veterinary visit (32). There was a decrease in owner-reported anxiety-related behaviors, such as stranger-directed fear, non-social fear, and touch sensitivity following a 7-week feeding period.

The primary objective of the present study was to explore the effect(s) a single dose of CBD in combination with L-tryptophan and *α*-casozepine had on measures of stress in dogs exposed to car travel when compared to other doses of CBD alone and a placebo. It was hypothesized that CBD, L-tryptophan, and α-casozepine administered in combination may provide an enhanced effect on reducing measures of stress in dogs. The secondary objectives of the project were to replicate previous findings related to the efficacy of CBD dosed at 4 mg/kg BW in reducing stress in response to car travel, and to explore the efficacy of a lower dosage of CBD at 2 mg/kg BW.

## Materials and methods

### Powering

The minimum sample size was determined using a powering by simulation for the primary measure of a change in cortisol levels from baseline to post-test. Data were simulated for four treatment groups based on parameters from existing data from dogs from the same facility and exposed to the same stress paradigm (4) with a meaningful difference in change in cortisol (10 ng/mL) induced in one treatment group. A linear mixed effects model was fitted on the simulated data, with change in cortisol as the response variable, treatment group as the fixed effect, and individual dog as the random effect. Contrasts between all treatment groups were made and *p*-values were then obtained. Power was calculated as the number of times a significant result was returned for all comparisons between the treatment group with the induced effect and the other treatment groups (*p* ≤ 0.05) divided by the total number of times the simulation was run (*n* = 1,000). Results indicated a minimum sample size of 48 dog was required to achieve 80% power for detecting changes in cortisol. In order to account for potential dropouts and missing data, a total of 54 dogs were recruited.

### Animals and husbandry

This project was reviewed and approved by the Waltham Animal Welfare and Ethical Review Body (PPM 112833) and conducted under the authority of the Animals (Scientific Procedures) Act 1986. Fifty-four healthy, adult dogs from small (Norfolk Terrier; *n* = 19), medium (Beagle; *n* = 9) and large breeds (Labrador Retriever; *n* = 26) with a mean age of 3.6 years (min: 1.5, max: 6.7) were selected for this study. Thirty (55.6%) dogs were male (all neutered) and 24 (44.4%) female (21 spayed, 3 entire). Dog weights across the study period ranged from 3.6 to 7.9 kg (mean: 5.3 kg) for Norfolk Terriers, 10.3 to 18.0 kg (mean: 14.2 kg) for Beagles, and 21.6 to 36.0 kg (mean: 28.5 kg) for Labrador Retrievers. Full demographic information for dogs recruited to the study are available in Supplementary Table S1.

All dogs were pair-housed within kennels at the Waltham Petcare Science Institute (Leicestershire, United Kingdom) with free access to indoor and outdoor environments. Dogs were either born on-site (*n* = 16) or acquired from external breeders (*n* = 38) and brought to site at on average 8.4 (range: 8.0 to 9.3) weeks of age. Dogs were provided with comprehensive training and socialization programs, adjusted to the needs of the individual dogs as per the Institute’s standard pet keeping requirements. All dogs routinely receive training to facilitate collection of samples (i.e., blood draws). In addition, prior to the study, dogs were trained to walk up or onto a box or ramp (based on the dog’s individual preference) into a fixed crate within the car. The number of training sessions provided varied based on the emotional reaction and training progression of the individual dog, with all dogs being required to be comfortable and willing to enter the crate within the car prior to their scheduled test sessions. Dogs in this study varied in their previous exposure to this stress paradigm based on their participation in past research. Three dogs had had five previous exposures to the stress paradigm as part of two studies (4, 44) and an additional 33 dogs had a single previous exposure to the stress paradigm (44). The remaining 18 dogs were naïve to this stress paradigm; however, all dogs may have had additional exposures to travel in a car if required for off-site veterinary care.

### Treatments

Four treatments were utilized during this study: a placebo, a single dose of 2 mg/kg BW CBD (CBD_2), a single dose of 4 mg/kg BW CBD (CBD_4), and a single dose of 2 mg/kg BW CBD combined with a blend of L-Tryptophan and *α*-casozepine (CBD_2_Blend). The target dosage of α-casozepine was a minimum of 20 mg/kg BW based on a previously reported effective dose (32). Tryptophan was formulated to target 0.65 g/MCal when fed in combination with a nutritionally complete diet (0.49 g/MCal FEDIAF minimum). The quantity of each active ingredient in the final treat recipe is reported in Table 1.

**Table 1:** Details of treats manufactured to contain different combinations of CBD/Placebo with *α*-casozepine and L-Tryptophan (Trp) for different sized dogs (S-Small, M-Medium, L-Large).

Treat type	Size	Weight	Contents
CBD	S	3 g	8.48 mg CBD
	M	4 g	16.97 mg CBD
	L	4 g	28.28 mg CBD
CBD_Blend	S	3 g	8.48 mg CBD; 45 mg Trp; 60 mg α-casozepine
	M	4 g	16.97 mg CBD; 70 mg Trp; 120 mg α-casozepine
	L	4 g	28.28 mg CBD; 85 mg Trp; 180 mg α-casozepine
Placebo	S	3 g	0 mg CBD
	M	4 g	0 mg CBD
	L	4 g	0 mg CBD
Placebo_Blend	S	3 g	0 mg CBD; 45 mg Trp; 60 mg α-casozepine
	M	4 g	0 mg CBD; 70 mg Trp; 120 mg α-casozepine
	L	4 g	0 mg CBD; 85 mg Trp; 180 mg α-casozepine

**Figure 1:**
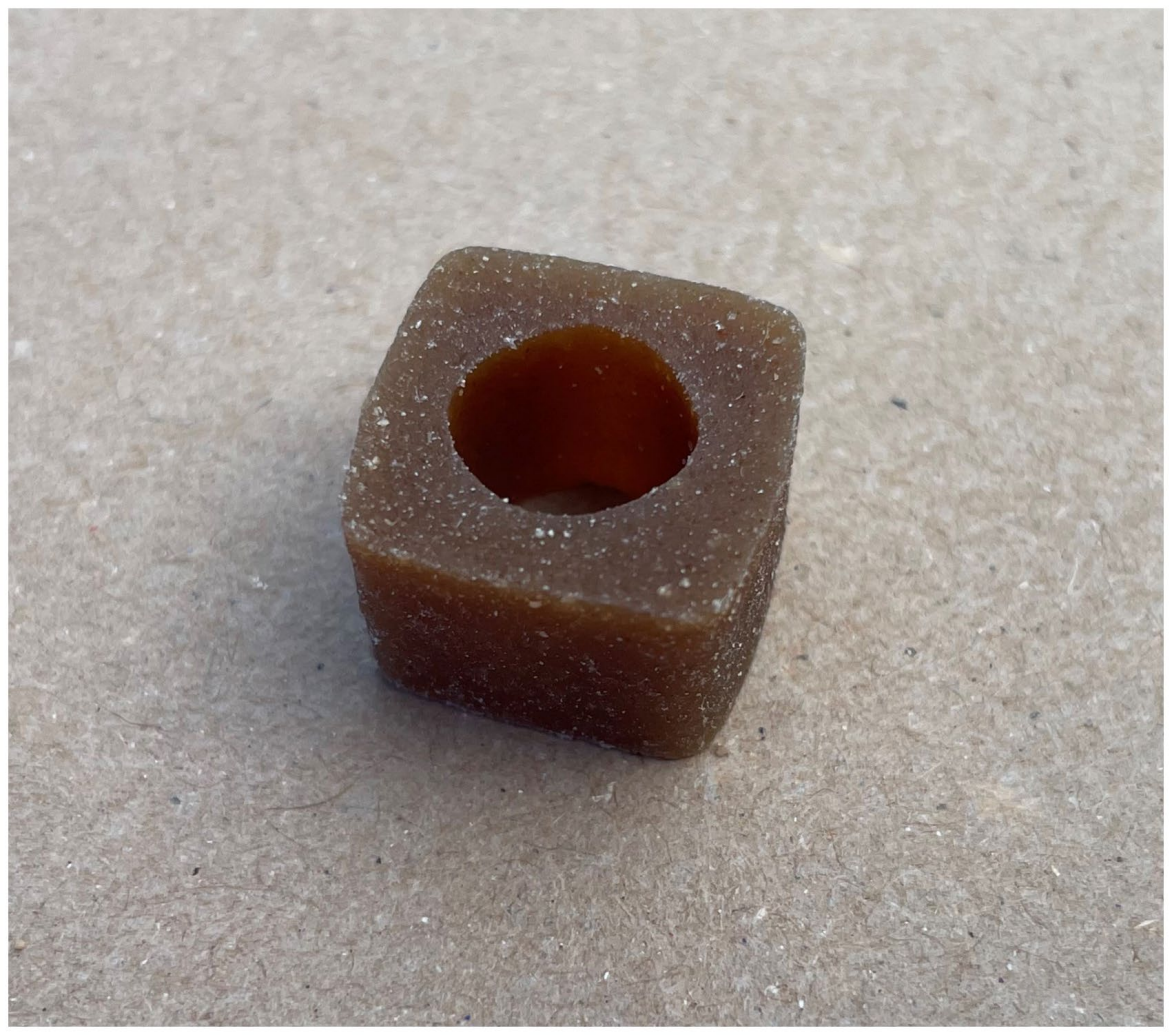
Semi-moist treat manufactured to include active ingredients for the delivery of the four different treatments: Placebo, CBD_2, CBD_2_Blend, CBD_4.

The relevant active ingredients (CBD, L-Tryptophan and *α*-casozepine) were incorporated into a treat recipe and extruded into a semi-moist treat format (Figure 1). The CBD used was a hemp-derived CBD distillate acquired from Kazmira LLC (Colorado, United States). The hemp-derived distillate was diluted with a food-grade sunflower oil to produce an oil with final concentration of 14.14% CBD. This CBD oil was analyzed by a third-party laboratory for full spectrum analysis of cannabinoid content (including CBD and THC), potential contaminants, and potency (Botanacor Laboratories, Colorado, United States). The THC content was below the limit of analytical detection (<0.02 mg/mL) and CBD was the only cannabinoid at detectable levels. The Placebo oil consisted of the same food-grade sunflower oil used within the CBD oil without inclusion of the CBD distillate.

In order to maintain blinding of study personnel, four different treat types were manufactured (Table 1) and were assigned unique codes using randomly generated numbers. These treat types were combined to generate each of the four treatments: Placebo (2x Placebo treats), CBD_2 (CBD + Placebo treats), CBD_2_Blend (CBD_Blend + Placebo_Blend treats), CBD_4 (2x CBD treats). Further, in order to support targeted dosing for dogs of different sizes, three different treat sizes were manufactured (small, medium, large). Other than differences in size for the small, medium and large treats all treat types were visually similar. The same base recipe was used for all treats, with the only difference in smell or taste being due to the inclusion of the active ingredients.

The quantity and size of treats to be given was determined based on a pre-defined feeding guide (Table 2), and the weight of the dog recorded in the week prior to treatment administration.

**Table 2:** Feeding guidelines for the different sized treats based on dog weight.

Dog weight	Treat size	Total treat number
<6 kg	Small	2
6–8 kg	Small	3
8–12 kg	Medium	2
12–16 kg	Medium	3
16–20 kg	Medium	4
20–27 kg	Large	3
27–34 kg	Large	4
34–40 kg	Large	5
>40 kg	Large	6

One of the final treat products (CBD Large 4 g) was tested for the presence of CBD and stability following storage for up to 12 months. The expected level of CBD based on 5% inclusion of the 14.14% CBD oil was 0.71%. Results of the stability analysis identified CBD present at 0.73% following 1 month of storage, and ranged between 0.61 and 0.67% for the remaining months up to 12 months.

### Diet

To control for the potential influence of diet on treatment metabolism, all dogs were maintained on a single batch of a standard commercial dry diet (Royal Canin® Medium Adult Dry, Royal Canin, Aimargues, France) for the duration of the study, including a 2 week prefeed prior to the start of testing. The diet underwent nutritional analysis (Eurofins, United Kingdom) and was found to meet essential nutrient requirements, and contained tryptophan in levels of 0.68 g/Mcal. Dogs also received a size appropriate dental chew (GREENIES™ Original Adult Dog Treats, Mars Petcare US, Franklin, TN) with their PM meal for daily dental care. Provision of any other food was restricted during the study period, with the exception of one Beagle that received high value food reinforcement to aid in additional study-related training sessions (no high value food reinforcement was provided during the 2 weeks immediately preceding a treatment).

### Study design

The study took place between April and August 2023, and utilized a blinded, balanced and randomized crossover design. The study consisted of four study phases with 4 weeks between the start of each phase. Each dog received four different treatments, one in each study phase, with treatment order randomly allocated based on a balanced Latin-square design. Five dogs had to skip their scheduled test session in at least one study phase due to identification of additional time required for training prior to testing (*n* = 2) or due to the dog refusing to voluntarily enter the car during their scheduled session (*n* = 3). The three refusals occurred following treatment with CBD_2 (*n* = 1) and CBD_4 (*n* = 2). These dogs continued with their scheduled session in the next study phase following additional training, resulting in 8 weeks between sessions. An additional session was then scheduled at the end of the study (2–4 weeks after previous session) for these dogs to receive their final treatment.

**Figure 2:**
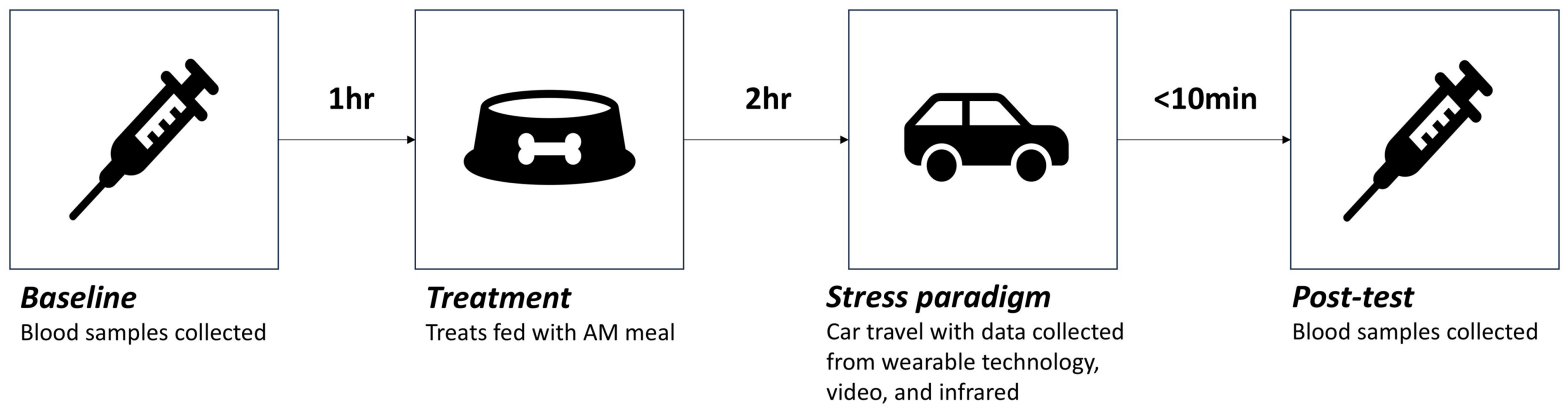
Timing and order of the different stages of test sessions where dogs were given one of four treatments (Placebo, CBD_2, CBD_2_Blend, CBD_4).

Test sessions consisted of a baseline blood sample (3 h prior to stress paradigm), followed by treatment administration (2 h prior to stress paradigm), the stress paradigm (car travel) and post-test sampling (within 10 min of the end of the stress paradigm; Figure 2). In order to minimize the impact of daily activities on dogs’ emotional responses and/or measures of stress, each dog was restricted from potentially impactful activities, such as walks and training sessions, until after test sessions on test days. Details of each of the timings and measures collected at each of these stages are presented below.

### Treatment administration

Dogs were given one of the four treatments immediately prior to their morning meal (08:30–10:45), with the timing of the meal scheduled to occur 2 h prior to the dog’s stress paradigm. A two-hour delay between dosing and testing was selected based upon reported peak CBD levels in the blood between 1.5 and 2 h after dosing (18, 22, 27). Dogs were offered the treats from a standard bowl, and acceptance was recorded after 5 min to ensure inclusion of the active ingredients did not impact palatability. All dogs consumed the treats within this time, with the exception of one individual who refused the treat (CBD_2_Blend) on initial offering but consumed all treats after they were soaked in warm water.

### Stress paradigm

On test days, dogs were exposed to a stress paradigm consisting of a 10-min car journey 2 h after treatment administration. The car journey was conducted in a minivan vehicle (Ford S-Max, Ford Motor Company Ltd., Essex, United Kingdom) with an XL black metal wire dog crate (106 × 71 × 70 cm; Ellie-Bo™, Stack m High Ltd., Weyhill, United Kingdom) containing a piece of non-slip vet bedding placed in the back of the vehicle on the folded down rear seats. Dogs were collected by a familiar handler and fitted with an optical heart rate (HR) monitor (Polar® Verity Sense Monitors, Polar Electro, Kempele, Finland) and activity monitor (Whistle FIT®, Mars Petcare, McLean, VA, United States) attached to a collar. After fitting the collar, dogs were taken outside and cued to enter the car via a ramp or box based on their individual preference and training. Dogs were then shut in the crate, the boot closed, and the car journey was initiated. If dogs did not voluntarily enter the car following trained cues and gentle encouragement (i.e., tapping back of car and calling name) the test session was terminated and rescheduled following additional training.

The car journey consisted of a range of maneuvres, including a sharp U-turn and a 3-point turn. The speed of the car never exceeded 10 mph due to being in a private enclosed car park. On completion of the journey, the dog was removed from the car by their handler and led to a room for post-test sampling. Throughout testing dogs were monitored for safety and signs of distress based upon pre-defined stop criteria. Distress was defined as a collection of stress-related behaviors occurring simultaneously over a prolonged duration and/or behaviors of intense magnitude and included self-mutilation, frantic destruction that may cause harm to the animal, excessive barking, fast respiration/hyperventilation, hypersalivation, cowering, and vigorous attempts to escape. No test sessions had to be halted due to concerns related to safety or distress during the study. Additional details on the stress paradigm have been previously reported (4, 5).

### Measures of stress

A number of behavioral and physiological measures were collected before, during and after the stress paradigm. In addition, blood parameters were collected at a baseline timepoint 1 h prior to treatment administration. Furthermore, infra-red thermography data were collected upon the dog’s entry and exit of the car; however, the results of this data are not reported.

#### Blood parameters

Blood samples were collected for each dog at baseline (1 h prior to treatment administration) and again after completion of the stress paradigm. All blood samples were collected within 10 min from entry into the sampling room to minimize the impact of any potential stress of sampling on the collected measures. Prior to the sample, a small patch of hair was shaved from the dog’s neck and a disinfectant wipe (Vetasept, Animalcare Ltd., York, United Kingdom) and topical anesthesia (Ethycalm Plus™, Invicta, West Sussex, United Kingdom) was applied to the area, then a 1.5 mL blood sample was collected from the jugular vein.

Blood was aliquoted into a clot-activating serum tube and left to stand for 30 min for analysis of serum cortisol, and into an EDTA tube and inverted 10 times then placed on rollers before being put on ice for analysis of plasma CBD. Blood samples were then transported to an on-site laboratory for processing. For serum cortisol, the tubes were spun down (centrifuge 2000 g for 10 min at ambient temperature), aliquoted, and frozen at −80°C until analyzed. For plasma CBD, the tubes were spun down (centrifuge 2000 g for 10 min at 4°C), aliquoted, and frozen at −80°C until analyzed. Cortisol analysis was performed using the R&D Systems, Parameter Cortisol Immunoassay (bio-techne, Minneapolis, United States) following the manufacturer’s protocol with an intra-assay variation of <10%. Analysis of plasma CBD was performed using previously reported methods (19, 20) using an Agilent 1,290 liquid chromatograph coupled with a 6,460 Triple Quadrupole mass spectrometer, operated by Masshunter software (Agilent; United States). The lower limit of detection (LOD) after correction for dilution was 3 ng/mL CBD, and the lower limit of quantification (LOQ) was 12 ng/mL.

#### Wearable technology

Dogs wore HR monitors (Polar® Verity Sense) attached to a collar (separate to that individual’s identification collar) throughout the stress paradigm in order to collect HR data. The monitors were positioned on the dog’s neck over the jugular vein, where hair had previously been shaved in preparation for blood sampling. Data were filtered to include only the period of time the dog was in the stress paradigm and summarized to obtain the mean and maximum HR recorded. In addition, heart rate variability (HRV) was determined as the root mean square of successive differences between normal heartbeats (RMSSD) by estimating the between-beat time differences from the HR measurements.

Additional data were obtained from activity monitors (Whistle FIT), which were attached to the dog’s identification collar. One-minute Activity Points were calculated based on the raw activity data and were indicative of the duration and intensity of activity during that time period. These Activity Points were then filtered to include only the period of time the dog was in the stress paradigm and summarized to determine mean Activity Points recorded.

#### Video footage

Video footage of the dogs was collected during the stress paradigm using two webcams (Logitech 922 Webcam, Logitech, Lausanne, Switzerland). One webcam was mounted to the central console of the car, facing backwards for a view of the front of the crate and the other was attached to the rear window of the car facing forwards for a view of the back of the crate. Videos were recorded using Media Recorder program (Noldus, Netherlands, Europe) and lasted for the duration of the 10-min test session. A number of behaviors anticipated to be related to stress and anxiety during car travel were coded using a detailed ethogram (Table 3) and The Observer XT 15 (Noldus, Netherlands, Europe) coding software. Behaviors were coded continuously during the 10-min test session, with coding starting once the coder could see the car journey had initiated, based on seeing external movement in the car’s rear window, and ending when the coder could clearly see the car had come to a complete stop at the end of the journey. Durations were coded for state behaviors, and counts were coded for event behaviors.

**Table 3:** Ethogram used to identify behaviors relating to stress and anxiety that may be performed by dogs during the stress paradigm.

Grouping	Behavior	Definition	Coding type	Reference
Body position	Lie – Lateral	Side of dog touching the ground fully, both hind limbs on the same side of body.	State	Modified from Ley et al. (72)
	Lie – Sternal	Stenum touching ground, hind limbs on either side of body (bend or stretched out the back).	State	Modified from Ley et al. (72)
	Lie – Combination	Sternal in front and lateral behind; sternum touching ground, both hind limb on same side of body	State	Modified from Ley et al. (72)
	Sit	Fron legs straight, read end lowered and resting on hocks and perineum. Hind legs may be turned to one side.	State	Ley et al. (72)
	Stand	Upright on all four legs, or hind legs only.	State	Modified from Ley et al. (72)
Movement	Moving	Takes one or more steps in any direction by lifting legs in a controlled way while in a standing, sitting, or lying position (excluding uncontrolled movement related to loss of balance).	State	Modified from Skånberg et al. (73)
	Not Moving	Movement ceases, dog does not move legs.	State	Defined for this study
Bracing	Brace	A crouching body posture while standing, sitting, or lying with a low center of gravity. Tense legs, often in a wide angle and with spread nails. The distance between belly and floor is half the length of the legs or shorter.	State	Skånberg et al. (73)
	Not Bracing	Bracing ceases – dog resumes normal posture.	State	Defined for this study
Ear carriage	Neutral/Erect	Ears relaxed and held to the side of the head or pointed forward.	State	Flint et al. (74)
	Back	Ears pulled back against head.	State	Flint et al. (74)
	Ears Not Visible	Ears not visible due to camera angle or video quality.	State	Defined for this study
Panting	Panting	Increased shallow respiration through an open mouth, may have tongue out.	State	Hunt et al. (5)
	Not Panting	Mouth is closed - normal breathing resumes.	State	Hunt et al. (5)
	Mouth Not Visible	Mouth area not visible due to camera angle or video quality.	State	Defined for this study
Whining	Whining	Dog produces sounds such as whines, whimpers, yelps etc. originating from the throat and mouth.	State	Hunt et al. (5)
	Not Whining	Sound production ceases.	State	Hunt et al. (5)
Lip licking	Lick Licking	Dog flicks its tongue around the outside of its mouth, on lips and/or quickly over its nose.	Event	Hunt et al. (5)

A total of four coders were utilized for the coding of behavior. One trained dog behavior coder (Coder 1) scored all videos for the behavioral groupings of body position, movement, and bracing. Videos for the remaining behavioral groupings of ear carriage, panting, whining and lip licking were divided between three different dog behavior coders (Coder 2, 3 & 4). All coders received training in coding the behaviors of interest, including reviewing example video footage and discussing the ethogram. Prior to coding study videos, all coders scored 10 videos of dogs traveling in cars and were required to meet moderate inter-rater reliability agreement (ICC > 0.50) before continuing to study footage. If coders did not meet this level of agreement additional training was provided.

For the purposes of analysis, behaviors that were not always visible (due to the head of the dog being out of frame), such as panting and ear carriage, were converted to proportions by dividing the duration of time the dog was performing that behavior by the total video duration minus the duration of time the head was not visible. Any videos where a behavior could not be coded reliably for more than 25% of the total video duration were excluded from the analysis for that behavior. This resulted in 21 videos being excluded for panting, and 19 videos for ear carriage. Further, bracing was removed from analysis due to infrequent occurrence (mean duration: 6.3 s). To check for potential differences in excluded data caused by treatment, the probability of data being excluded for each behavior was compared between treatment groups and no significant effects were identified. Lying behaviors were analyzed as “sternal” and “not sternal” (Lie – Combination + Lie – Lateral) based on the hypothesis that “not sternal” lying positions would be indicative of more relaxed states.

For scoring of qualitative behavior assessment (QBA) a list of terms was generated (Table 4) by reviewing terms used in previous research (5, 44–47). Additional terms were added as needed after a random selection of videos were reviewed by the study team. The following terms were selected in order to capture the range of behaviors and emotions displayed by dogs: alert, anxious, apathetic, comfortable, curious, excited, fearful, frustrated, happy, restless, sad, tense.

**Table 4:** Definitions of the terms utilized during qualitative behavior assessment (QBA).

Term	Description	Reference
Alert	Vigilant, on guard, attentive, reactive to stimuli	Modified from Arena et al. (45)
Anxious	Worried, apprehensive, unable to settle or cope with the environment	Modified from Arena et al. (45)
Apathetic	Uninterested, indifferent, absence of positive or negative emotion, non-responsive to external stimuli	Defined for this study
Comfortable	Without worry, settled within environment, relaxed, peaceful with external stimuli	Modified from Arena et al. (45)
Curious	Actively interested in stimuli, explorative, interested, inquisitive	Modified from Arena et al. (45)
Excited	Euphoric, exuberant, positive heightened arousal in response to external stimuli	Modified from Arena et al. (45)
Fearful	Scared, timid, afraid, terrified, shows postures typical of fear	Modified from Arena et al. (45)
Frustrated	Annoyed, irritable, unable to obtain what it wants, impatient	Flint et al. (46)
Happy	Delighted, pleased, joyful, content	Flint et al. (44)
Restless	Unable to settle or relax, hyperactive	Modified from Hunt et al. (5)
Sad	Unhappy, downcast, depressed, dull, disengaged	Modified from Flint et al. (46)
Tense	Stiff, rigid posture, on edge	Flint et al. (46)

One dog behavior coder (Coder 1) provided scores on all videos collected during this study. This coder had extensive previous experience with scoring dog behavior using QBAs from previous studies within the research group (4, 5, 44, 46). Additional discussions were held between the coder and the study team, including reviewing existing video footage of dogs traveling in cars and aligning on interpretation of the scale and terms used. During scoring the coder was instructed to watch the 10-min video, then provide one score per term. Terms were scored using a visual analog scale (without numerical anchors), where the left end of the scale indicated the dogs were expressing a total lack of, or negligible amount, of the attribute indicated by the term, and the right end of the scale indicated the dog was strongly expressing the attribute indicated by the term. The location of the slider on the visual analog scale was then converted to a numerical score from 0 to 124.

For all video coding, a random selection of 10 videos were re-coded by the same coder for a total of three repetitions for intra-rater reliability analysis. These videos were the same for all coders and were also used for inter-rater reliability analysis for behaviors with multiple coders. Video order was randomized, and video names were encoded so that the coders were blind to which videos were from which test sessions, as well as which videos were included in reliability analysis.

### Statistical analysis

All analyses were performed using R Statistical Software version 4.2.2 (48).

To assess the effect of treatment on post-test CBD plasma levels a linear mixed effect model [via “lme4” R packages; (49)] was fitted. Post-test CBD plasma level was the response variable, with treatment as the fixed effect, and individual animal nested within breed included as the random effect. For the effect of treatment related to CBD absorption, pairwise comparisons were made between all groups with a Tukey test, with simultaneous multiple comparisons correction implemented through the glht ‘single-step’ method [via “multcomp” R package; (50)]. Model assumptions for normality of residuals were assessed visually using histograms and Q-Q plots, while assumptions of homoscedasticity of residuals were assessed by generating a scatterplot of residuals against predicted values. Variables were log10-transformed if they violated model assumptions.

To assess the effect of treatment on measures of stress in dogs each of the individual measures were fit to separate linear mixed effect models [via “lme4” R packages; (49)] with the individual measure as the response variable, treatment as the fixed effect, and individual animal nested within breed, and study phase as the random effects. Model assumptions for normality of residuals were assessed visually using histograms and Q-Q plots, while assumptions of homoscedasticity of residuals were assessed by generating a scatterplot of residuals against predicted values. Variables were log10-transformed if they violated model assumptions, with a small constant (0.01) added to allow for transformation of zero values where required. The estimated means (back-transformed and constant subtracted for log-transformed models) and 95% CI were extracted from the models. Pairwise comparisons were made between each treatment group against the placebo group with a Dunnett test, with simultaneous multiple comparisons correction implemented through the glht ‘single-step’ method [via “multcomp” R package; (50)]. For each pairwise comparison, the estimated differences (fold-changes for log-transformed models), 95% CI, and *p*-values were obtained and significant effects (*p ≤* 0.050) were reported.

Infrequent behaviors (>25% of observations scored as 0) were converted to 0/1 (indicating the absence/presence of the behavior) and analyzed using logistic regressions [via “lme4” R packages; (49)], with the same fixed and random effects as described above. Estimated predicted probability and 95% CI were extracted from the models. For each pairwise comparison the odds ratios, 95% CI, and p-values were obtained and significant effects (*p ≤* 0.050) were reported.

Following observations that plasma CBD levels were widely variable between dogs, the linear and logistic mixed effect models were repeated with the individual measures as the response variables, but with post-test CBD plasma levels and whether the dog was given the blend as the fixed effects. An interaction between CBD plasma levels and presence of blend was also tested. The same random effect structure was used as defined for the models examining the effect of treatment. Model assumptions for normality of residuals were assessed visually using histograms and Q-Q plots, while assumptions of homoscedasticity of residuals were assessed by generating a scatterplot of residuals against predicted values. Variables were log10-transformed if they violated model assumptions, with a small constant (0.01) added to allow for transformation of zero values where required. The estimated means (back-transformed and constant subtracted for log-transformed models) and 95% CI were extracted from the models. The estimated changes per 100 ng/mL increase in plasma CBD (fold-changes for log-transformed models), 95% CI, and *p*-values were obtained and significant effects (*p ≤* 0.050) were reported.

A principal components analysis (PCA) of all the QBA terms was conducted using R package “FactoMineR” (51). The weightings from this analysis were used to generate individual scores for each of the relevant identified components, which were modeled as above using linear mixed-effects models to determine the effect of treatment. Prior to the PCA being conducted, the suitability of data for inclusion was tested using the ‘performance’ R package (52).

Reliability of the relevant individual PCA component scores as well as the coded behaviors were calculated using intraclass correlation coefficients (ICCs) from two-way mixed effects models using the R package ‘irr’ (53). Consistency agreement was used for inter-rater reliability (where relevant) and absolute agreement was used for intra-rater reliability (54). These values were interpreted as poor (ICC < 0.50), moderate (ICC: 0.50–0.75), good (ICC: 0.75–0.90) or excellent (ICC > 0.90) (54). Coders were required to meet a minimum of moderate reliability for the data to be included in further analysis.

## Results

### Qualitative behavior assessment PCA

**Figure 3:**
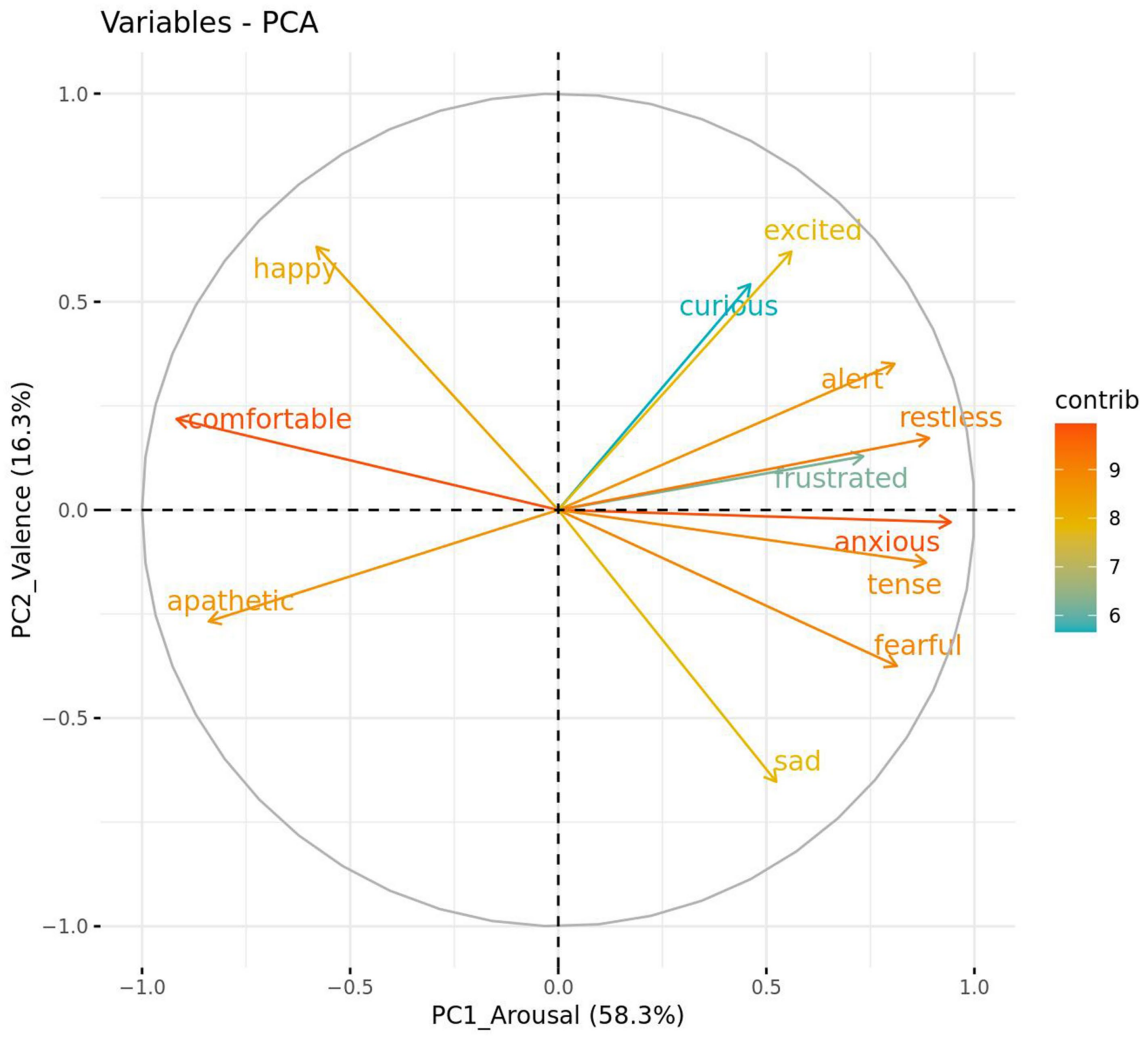
Strength and direction in which qualitative behavior assessment (QBA) terms loaded against two PCA component scores, PC1_Arousal and PC2_Valence. Bracketed numbers represent the total variance explained by each component score.

The QBA data met the requirements of a Kaiser–Meyer–Olkin (KMO) measure of sampling adequacy with KMO values >0.50 (overall KMO = 0.89) and a significant Bartlett’s test of sphericity (*p* < 0.001) (55). Analysis of the QBA data using a PCA suggested two main components of interest based on the strength of loadings, the variance explained, and eigenvalues greater than one (Figure 3). The first component explained 58.3% of the total variance and was labeled “PC1_Arousal.” It was comprised of positive loadings (> 0.60) for the terms anxious, restless, tense, fearful, alert, frustrated and negative loadings (< −0.60) for the terms comfortable and apathetic. The second component explained 16.3% of the total variance and was labeled “PC2_Valence.” It was comprised of positive loadings for the terms happy and excited, and negative loadings for the term sad.

### Rater reliability

Pre-study alignment demonstrated moderate to excellent reliability for all coded behaviors (ICC 0.71–0.99). Inter-rater reliability of the study footage was excellent for lip licking (ICC 0.97) and panting (ICC 1.00) behaviors, and poor for ear carriage (ICC 0.48). Whining had moderate inter-rater reliability (ICC 0.58), however this was primarily driven by one outlying value (entire video duration coded as whining), which was identified as a potential coding error. When this observation was removed whining had excellent inter-rater reliability (ICC 0.97).

Coder 2 had excellent intra-rater reliability for whining (ICC 0.97), lip licking (ICC 0.99) and panting (ICC 1.00) and moderate reliability for ear carriage (ICC 0.54). Coder 3 had excellent intra-rater reliability for panting (ICC 0.99) and whining (ICC 0.98), and good reliability for lip licking (ICC 0.88) and ear carriage (ICC 0.86). Coder 4 had excellent intra-rater reliability for panting (ICC 1.00) and lip licking (ICC 0.99), good reliability for ear carriage (ICC 0.77), and poor reliability for whining (ICC 0.13). However, as noted previously, this was largely driven by one outlying value, which was identified as a potential coding error. When this observation was removed Coder 4 had excellent reliability for whining (ICC 0.97). The poor reliability for ear carriage was discussed with the coders and was determined to be likely due to issues with video quality, such as variable background lighting while the car was in motion, and poor visibility of the ears, especially in dark colored dogs. Therefore, further training was not deemed to be beneficial and instead this behavior was excluded from further analysis.

Due to the body position and movement behaviors being coded by only a single rater inter-rater reliability was not calculated. The results for intra-rater reliability indicated excellent agreement for the behaviors moving, sitting, standing, and lying (ICC > 0.90), good agreement for lying in a “not sternal” position (ICC 0.86), and moderate agreement for lying in a “sternal” position (ICC 0.62).

Similarly, QBA scores were provided by a single rater, therefore inter-rater reliability was not calculated. Results for intra-rater reliability indicate that agreement was moderate of PC1_Arousal (ICC 0.62), and poor for PC2_Valence (ICC 0.33). Due to the poor reliability for PC2_Valence, only PC1_Arousal was included in further analyses.

### Treatment dosages

Due to the feeding guide being designed to minimize exceeding the target doses of CBD, actual dosages of CBD given to dogs during the study were slightly under the targets of 2 and 4 mg/kg BW. The true mean CBD dosage provided to dogs was 1.8 mg/kg BW (range: 1.4–2.4 mg/kg BW) for the CBD_2 and CBD_2_Blend treatments, and 3.6 mg/kg BW (range: 2.8–4.6 mg/kg BW) for the CBD_4 treatment. Dosages of the additional active ingredients were on average 14.3 mg/kg BW (range: 10.0–24.1 mg/kg BW) for L-tryptophan and 24.2 mg/kg BW (range: 21.2–32.1 mg/kg BW) for *α*-casozepine.

### CBD absorption

All dogs had plasma CBD levels below the LOD (<3 ng/mL) at baseline and when given the placebo treatment, except for one session where a dog had levels of 3.32 ng/mL at baseline on their final treatment (CBD_4). There were also 13 (8.8%) test sessions where dogs had CBD levels below the LOD after treatment with CBD, with an additional 15 (10.2%) dogs having values below the LOQ (<12 ng/mL). This included seven dogs (13.7%) below LOD when given CBD_2, three dogs (6.4%) when given CBD_2_Blend, and three dogs (6.1%) when given CBD_4. Three dogs (2 NT, 1 BE) did not have detectable absorption of CBD for two of the three treatments, and seven dogs (5 NT, 1 BE, 1 LR) did not have detectable absorption of CBD for only one treatment. For the purposes of analysis, values below the LOD were imputed as LOD/2 (1.5 ng/mL), and values between the LOD and LOQ (3–12 ng/mL) were included as estimated concentrations in order to minimize potential bias (56).

**Figure 4:**
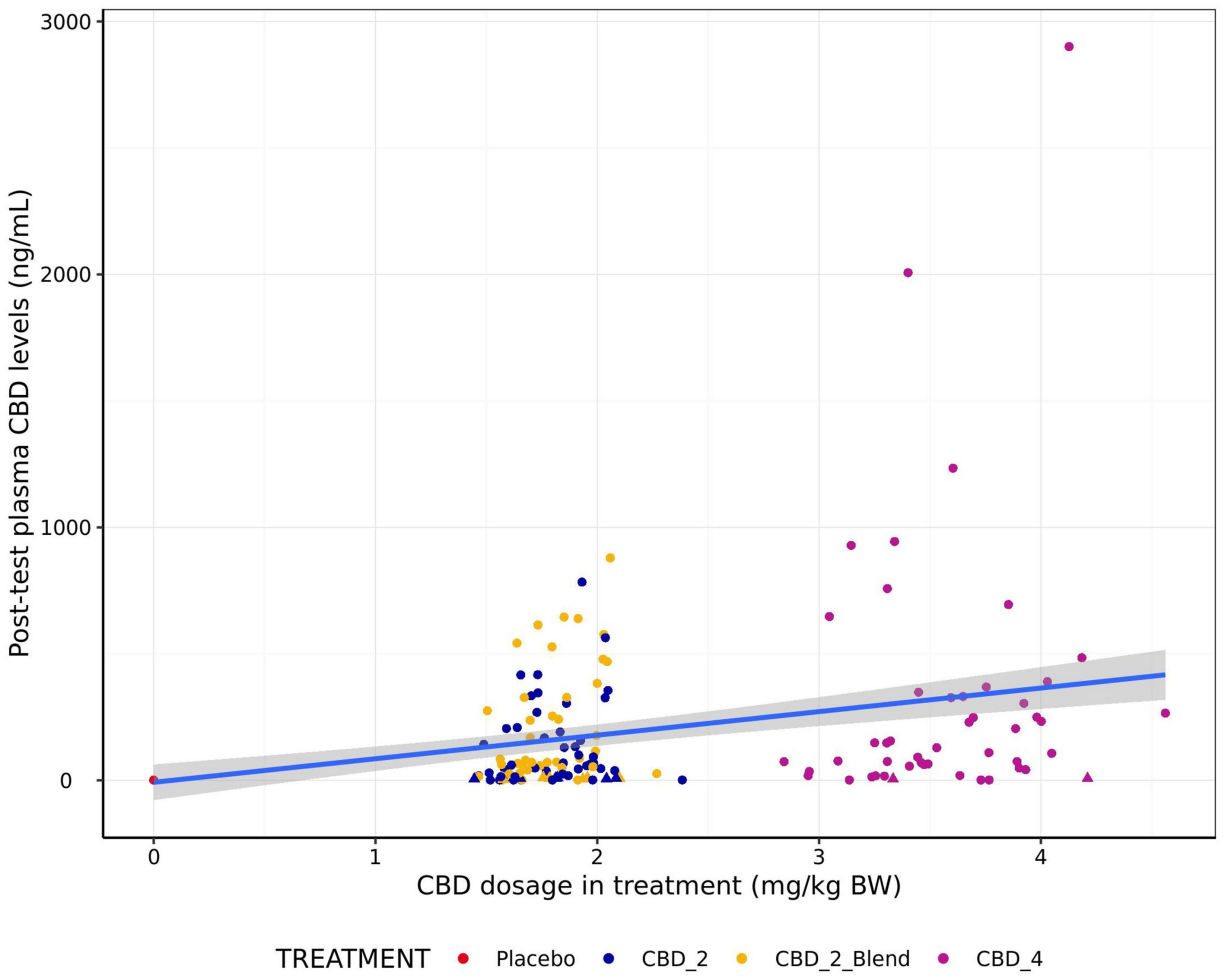
Relationship between post-test blood plasma CBD levels (ng/mL) and CBD dosage in treatment (mg/kg BW) for all four treatments. Points in triangle shape indicate estimated concentrations between the LOD (3 ng/mL) and LOQ (12 ng/mL) and values below LOD are imputed as LOD/2 (1.5 ng/mL). Blue line indicates a line of best fit generated using a linear model with gray shading indicating 95% confidence intervals.

Visual comparison of CBD plasma levels against actual treatment dosages indicated plasma levels increased with increasing dosages (Figure 4). However, a high degree of variation, including a large proportion of dogs with low absorption was observed.

**Figure 5:**
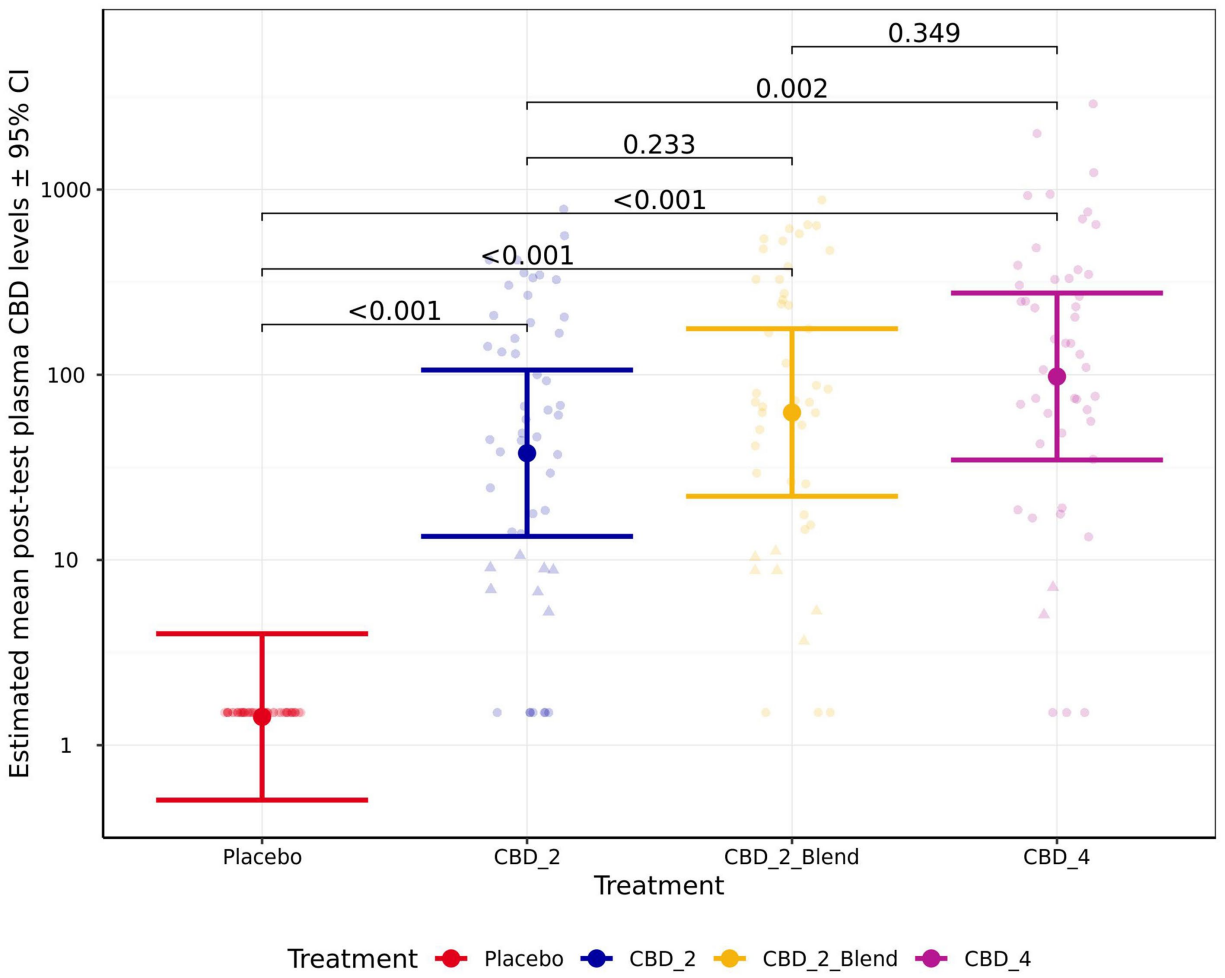
Estimated mean (back transformed) post-test blood plasma CBD levels (ng/mL) shown for all four treatments. Y-axis ticks placed on log scale. Error bars indicate ± 95% confidence intervals. Points in triangle shape indicate estimated concentrations between the LOD (3 ng/mL) and LOQ (12 ng/mL) and values below LOD are imputed as LOD/2 (1.5 ng/mL).

The model for CBD absorption demonstrated heteroscedasticity in the residuals and was log10 transformed, which improved model fit. Post-test CBD plasma levels were significantly associated with treatment (*p* < 0.001; Figure 5). Pairwise comparisons indicated that all the CBD treatments resulted in significantly higher post-test CBD plasma levels in comparison to the placebo (*p* < 0.001) and CBD_4 resulted in higher concentrations than CBD_2 (Fold Change: 2.59 [1.31, 5.13]; *p* = 0.002). However, no significant differences between the CBD treatments with and without the blend were identified: CBD_2 vs. CBD_2_Blend (*p* = 0.233); CBD_2_Blend vs. CBD_4 (*p* = 0.348). Estimated mean CBD plasma levels [±95% CI] were 37.72 ng/mL [13.41, 106.10 ng/mL] when dogs were given CBD_2, 62.56 ng/mL [22.07, 177.30 ng/mL] when dogs were given CBD_2_Blend, and 97.87 ng/mL [34.67, 276.30 ng/mL] when dogs were given CBD_4.

The random effects of individual animal (ICC 0.11) and breed (ICC 0.19) also contributed variability to the model of CBD plasma levels, with Norfolk Terriers demonstrating lower plasma levels in comparison to Labrador Retrievers.

### Treatment effect

The models for moving, sitting, standing, lip licking and panting demonstrated heteroscedasticity in the residuals and were log10 transformed improving model fit. Due to the presence of zero values, a small constant (0.01) was added prior to transformation. The models for lying, lying in a “sternal” position, and lying in a “not sternal” position also showed heteroscedasticity in the residuals, but log transformation did not improve model fit and therefore these models proceeded without transformation. All other models met the assumptions of normality and heteroscedasticity of residuals and proceeded without transformation. Whining demonstrated infrequent occurrence (>25% of observations scored as 0) and was converted to 0/1 (indicating the absence/presence of the behavior) and was analyzed using a mixed effects logistic regression.

**Figure 6:**
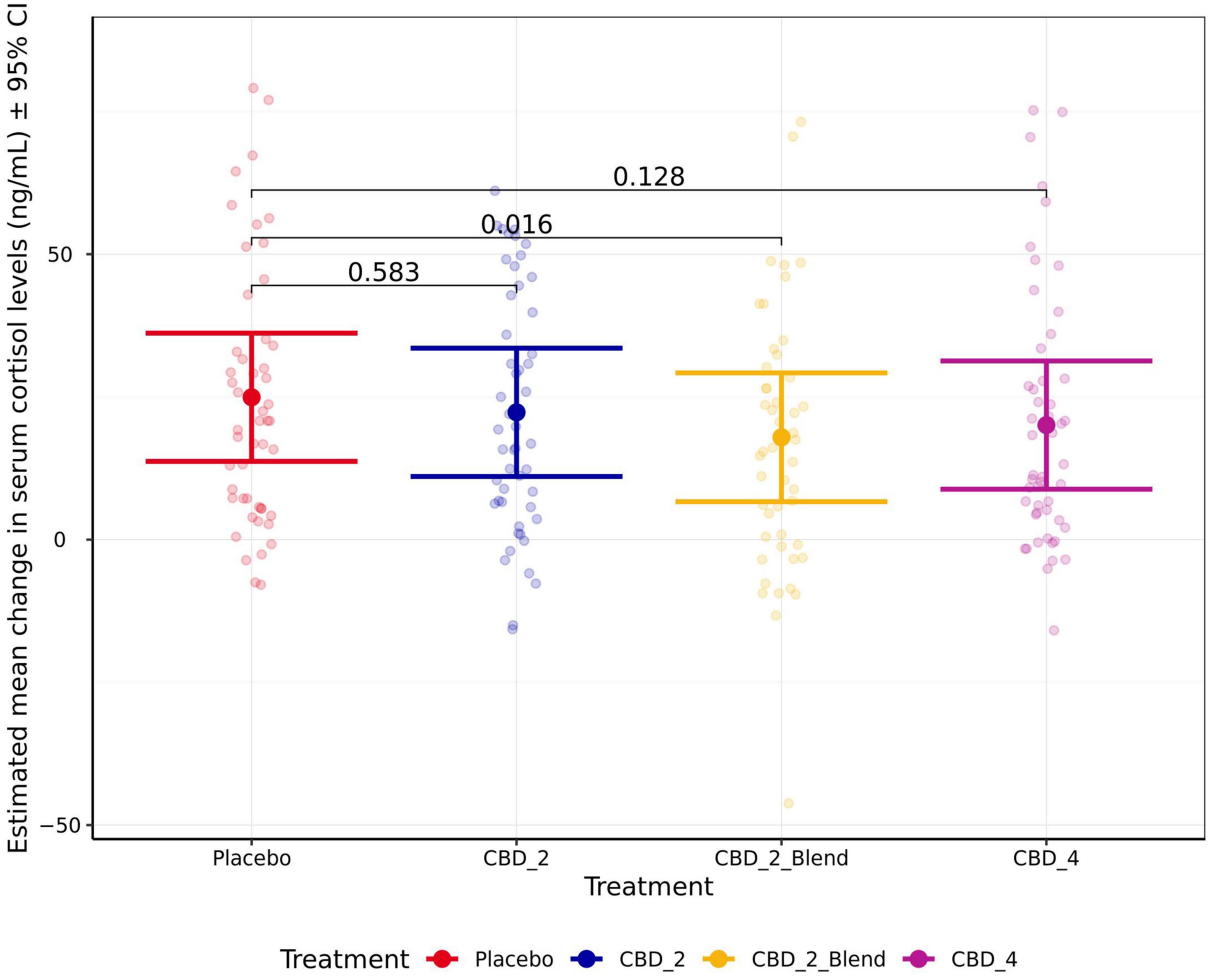
Estimated mean change in blood serum cortisol levels (ng/mL) shown for all four treatments. Error bars indicate ± 95% confidence intervals.

Treatment had a significant effect on the change in cortisol from the baseline to the post-test timepoints (Figure 6), resulting in a smaller increase in cortisol when dogs were given CBD_2_Blend in comparison to when dogs were given Placebo (−7.01 ng/mL [−12.98, −1.05 ng/mL]; *p* = 0.016). All other treatments were not significantly associated with change in cortisol when compared to Placebo (*p* > 0.050). Individual animal differences (ICC 0.58) and study phase (ICC 0.12) also contributed variability to the model for cortisol, with dogs having a larger increase in cortisol after their first exposure to the stress paradigm.

**Figure 7:**
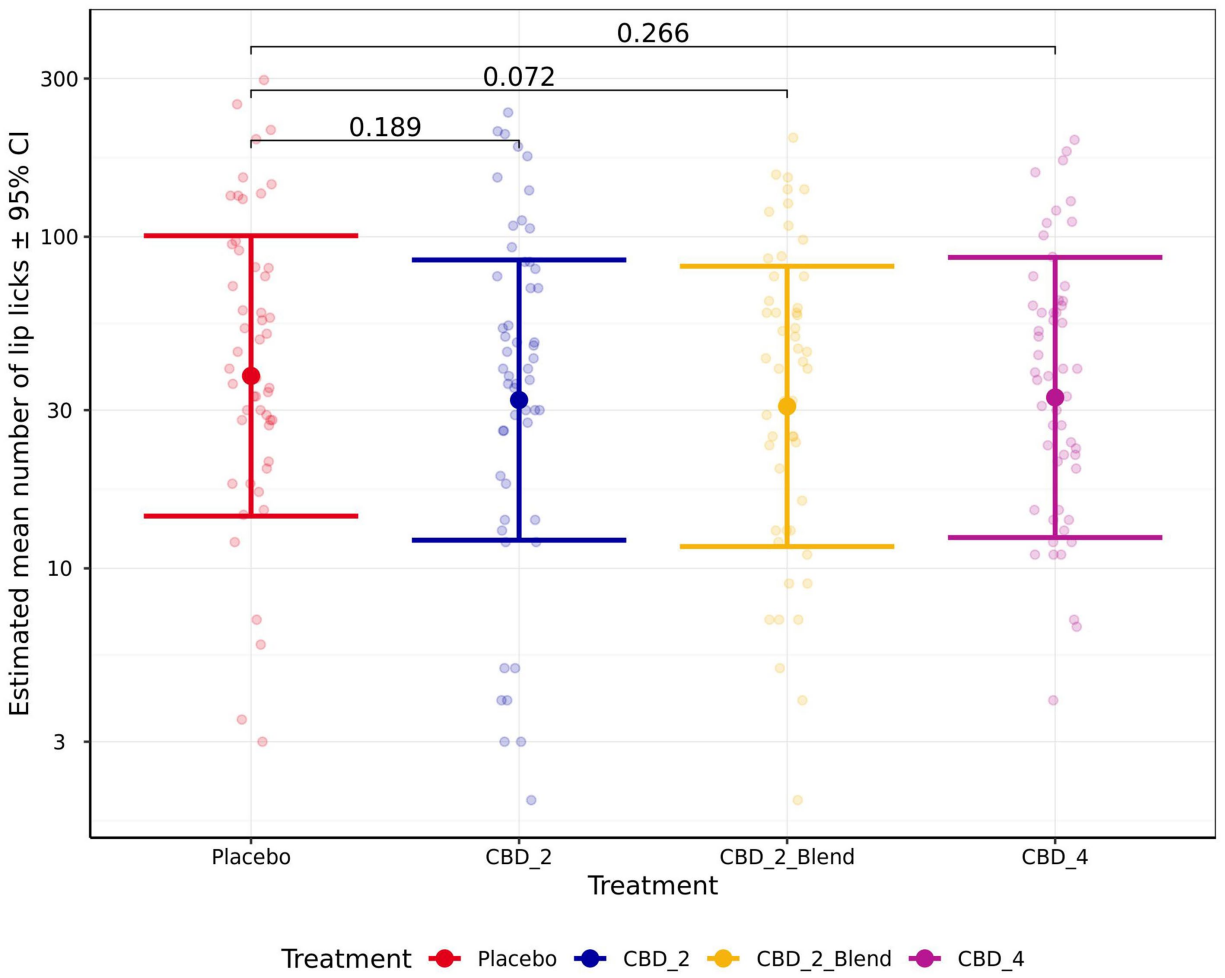
Estimated mean (back transformed) number of lip licking events observed shown for all four treatments. Y-axis ticks placed on log scale. Error bars indicate ± 95% confidence intervals.

**Figure 8:**
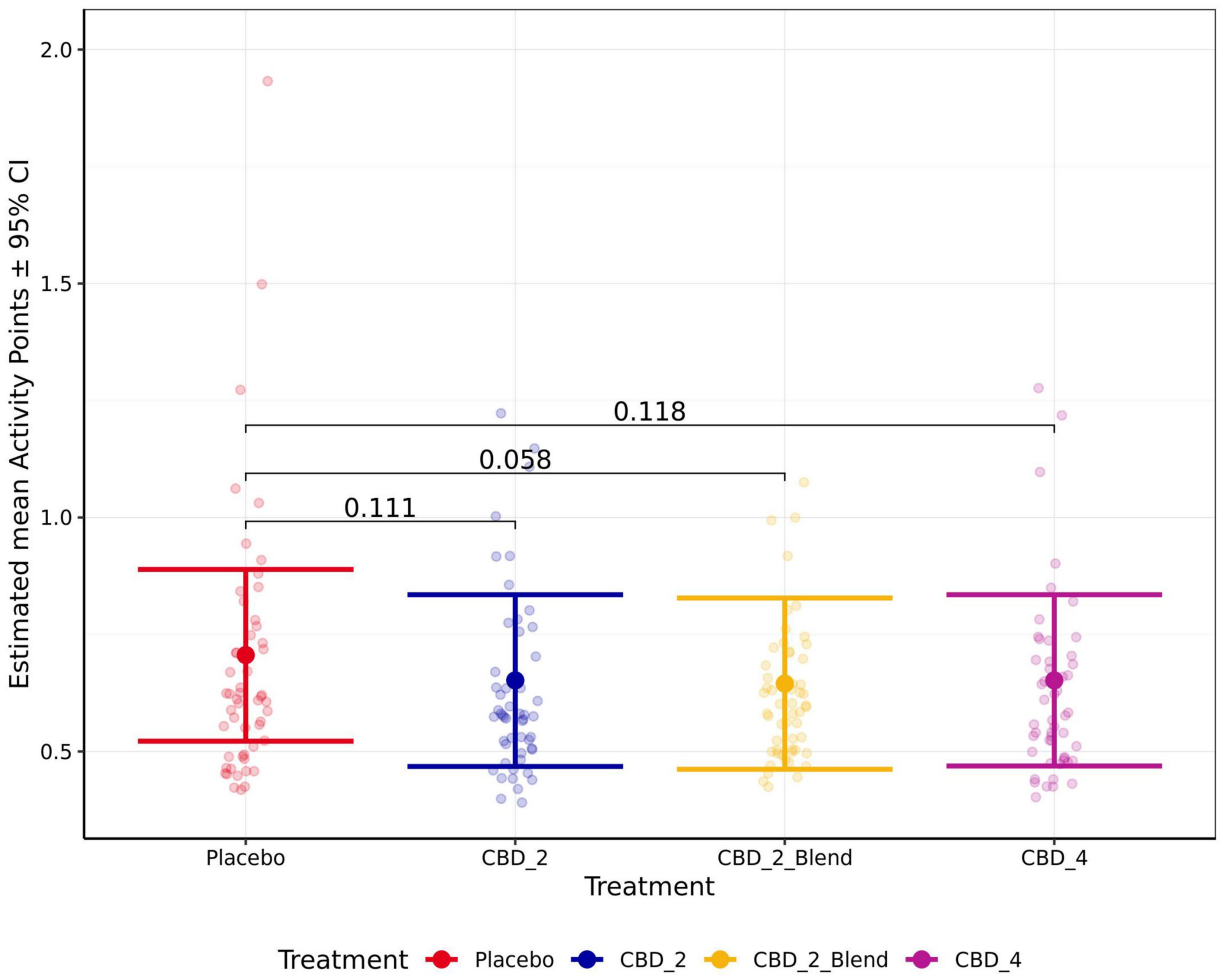
Estimated mean activity points shown for all four treatments. Error bars indicate ± 95% confidence intervals.

There were no significant effects of treatment on the remaining measures of HR, HRV (RMSSD), sitting, standing, lying, lying in a “sternal” position, lying in a “not sternal” position, moving, whining, lip licking, panting, Activity Points, or QBA PC1_Arousal ratings (p > 0.050). However, there was a non-significant trend for dogs to perform less lip licking (Fold Change: 0.81 [0.65, 1.01]; *p* = 0.072; Figure 7) and have lower Activity Points (−0.06 [−0.12, 0.00]; *p* = 0.058; Figure 8) when given CBD_2_Blend in comparison to when dogs were given Placebo.

### CBD plasma level effect

As with the models for treatment effects, the models for the effect of CBD plasma levels on moving, sitting, standing, lip licking and panting demonstrated heteroscedasticity in the residuals and were log10 transformed improving model fit. Due to the presence of zero values, a small constant (0.01) was added prior to transformation. The models for lying, lying in a “sternal” position, and lying in a “not sternal” position also showed heteroscedasticity in the residuals, but log transformation did not improve model fit and therefore these models proceeded without transformation. All other models met the assumptions of normality and heteroscedasticity of residuals and proceeded without transformation. Whining was analyzed using a mixed-effects logistic regression based on the presence/absence of the behavior and the continuous variable of post-test CBD levels was mean-centered and scaled for a 100-unit change in order to improve model convergence. The interaction between post-test CBD levels and presence of the blend was not significant for any measure and was therefore removed from the models.

**Figure 9:**
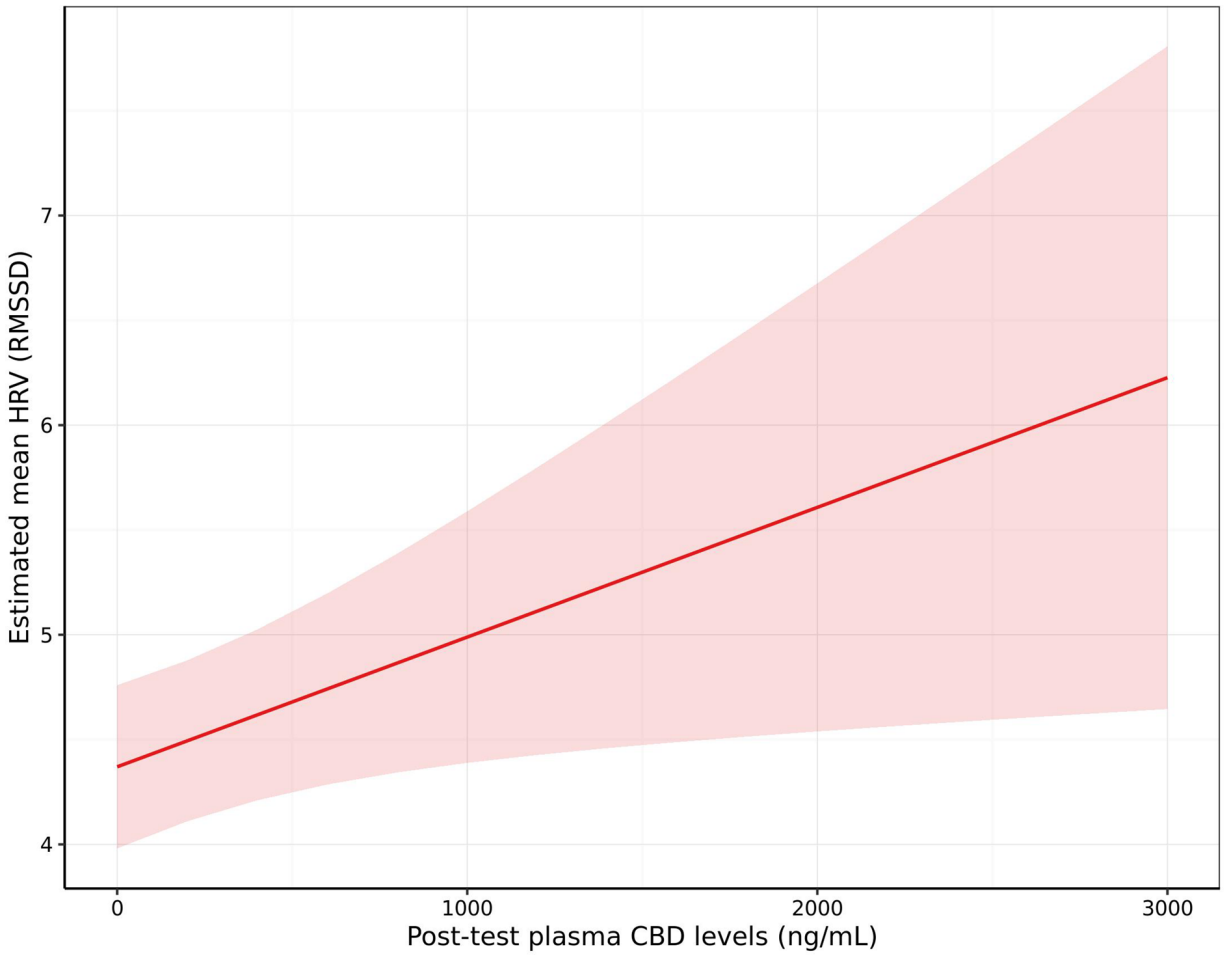
The effect of post-test blood plasma CBD concentration (ng/mL) on estimated mean heart rate variability (HRV) measured as root mean square of successive differences between normal heartbeats (RMSSD). Shading indicates the 95% confidence interval.

There was a significant effect of CBD plasma levels on HRV (RMSSD), with higher HRV observed in dogs with higher post-test CBD levels (+0.06 per 100 ng/mL CBD [0.00, 0.13]; *p* = 0.023; Figure 9). There were no significant effects of post-test CBD levels on the remaining measures, however there was a non-significant trend for whining to decrease (OR: 0.78 [0.56, 1.08]; *p* = 0.065) and standing to increase (Fold Change: 1.12 [0.97, 1.29]; *p* = 0.062) as post-test CBD levels increased. Additionally, there was a non-significant trend for the change in cortisol from baseline to post-test to be lower when dogs were given the blend in comparison to treatments without inclusion of the blend [−4.05 ng/mL (−9.09, 0.98); *p* = 0.055].

## Discussion

The aim of the present study was to determine the anxiolytic effect of a single treatment of CBD at two different dosages (2 and 4 mg/kg BW), as well as the effect of the inclusion of a blend consisting of L-tryptophan and *α*-casozepine on stress in dogs. Stress and anxiety were evaluated via a range of physiological and behavioral measures during and after exposing dogs to a brief period of car travel. Overall, the results indicated that while previous anxiety reducing effects of CBD at 4 mg/kg BW (4, 5) failed to be replicated with a different study population, there was a mild anxiolytic effect of CBD at 2 mg/kg BW with inclusion of the blend. Large individual variation in CBD absorption, as well as variation in response to the stressor likely limited the ability to identify significant effects of the treatments.

All CBD treatments resulted in significant increases in CBD plasma levels in comparison to the placebo, with the higher dosage of CBD at 4 mg/kg BW resulting in significantly higher CBD plasma levels in comparison to CBD at 2 mg/kg BW. While not significant, the levels of CBD in plasma were numerically higher when dogs were fed CBD in combination with the blend in comparison to the same dosage of CBD fed alone, suggesting the blend may have had an impact on CBD absorption or metabolism. Food provision has been previously shown to impact CBD absorption in humans [reviewed by Millar et al. (57)], and therefore specific attributes of the blend may have altered how CBD was absorbed.

Across the treatments, absorption of CBD was highly variable in the present study, with a number of dogs having CBD plasma levels below the lower limit of detection after treatment. Previous research by the authors has also demonstrated a high degree of variation in CBD plasma levels, both in samples collected 2 h after a single dose (4) and fasted samples collected after 2 or more weeks of treatment (20). Further, other published research has demonstrated a high degree of variation in absorption after a period of daily treatment (19, 58). However, all dogs in the previous studies did have detectable responses following treatment. It should be noted that a majority of these studies involved daily dosing, and therefore repeated dosing may have provided a cumulative effect, resulting in higher levels of plasma CBD being observed.

One other potential contributor to this difference in absorption may be the format in which the CBD was delivered. Previous studies administered CBD within oil either directly, or within a gel capsule. In the present study the CBD oil was combined with other ingredients and extruded into a treat format. While CBD was demonstrated to be present in expected quantities within the final treat product and did not show a meaningful decrease over 12 months while in storage, it is possible the manufacturing process limited the bioavailability of the CBD to the dogs, or altered the time required to reach peak plasma levels. Previous research looking at the effect of different preparations of CBD has demonstrated that provision in a semi-solid treat format results in lower peak levels and overall absorption in comparison to liquid preparation, although no difference in time to peak levels was observed (59). On the other hand (60), found the 24-h pharmacokinetics of CBD delivered in a soft chew to be similar to that delivered within an oil. Future studies may wish to consider exploring the *in vitro* digestibility of their CBD treatments in order to explore the suitability of their delivery matrix prior to testing.

As mentioned previously, different food attributes, such as fat content, have also been shown to affect bioavailability of CBD within human research [reviewed by Millar et al. (57)]. While diet was controlled between dogs within the current study, differences in diet, and the timing of meals may have contributed to different absorption levels observed in the current study in comparison to previous studies. However, within the current study dogs were observed with both outlying low as well as outlying high levels of CBD in the plasma following treatment, suggesting differences in bioavailability were not the sole cause of differences in absorption.

Feeding within a treat format also contributed to variation in actual dosing of CBD. As only half or whole treats could be fed with precision, dogs were given doses within a range based on a feeding guide, rather than exactly titred to their body weight. However, it appears individual variation also had a significant impact on CBD absorption, as even when exact CBD dose was plotted against CBD plasma levels there was not a strong linear relationship. Additionally, a number of dogs who did not have detectable absorption of CBD in response to one treatment also had low or non-detectable responses to the other treatments. Breed was found to impact CBD absorption, with Norfolk Terriers having lower CBD levels in comparison to Labrador Retrievers, an effect which was also observed within a previous study using the same dog breeds (20). Further research is warranted to understand what factors impact the absorption of CBD in dogs, including breed and size effects, as well as how to identify dogs who fail to adequately absorb CBD so that treatments can be appropriately targeted.

Due to the variability in CBD absorption, the efficacy of the treatments for reducing stress was analyzed in two different ways. First the relationship between measures of stress and the individual treatments of placebo, CBD at 2 mg/kg BW, CBD at 2 mg/kg BW with inclusion of the blend, and CBD at 4 mg/kg BW were analyzed. However, to better understand how CBD absorption impacted efficacy, the relationship between measures of stress and CBD plasma levels and presence of the blend was also analyzed.

Data indicated that dogs had a reduced stress response to the car travel stress paradigm when given the treatment of CBD at 2 mg/kg BW with inclusion of the blend in comparison to the placebo. This was demonstrated through a significant reduction in the change in cortisol from baseline to post-test. While they did not meet the threshold for statistical significance, and therefore should be interpreted with caution, there were also trends (*p* < 0.10) for reductions in lip licking and activity which may warrant further investigation. In addition to being considered as an indicator of stress, the performance of lip-licking behaviors while in a moving vehicle may also be interpreted as a symptom of motion sickness in dogs (61). While previous studies have shown a link between the endocannabinoid system and motion sickness (62, 63), a study by Cluny et al. (64) revealed the provision of CBD did not mitigate motion-related emesis in Asian house shrews (*Suncus murinus*). The other two treatments of CBD at 2 mg/kg BW without inclusion of the blend and CBD at 4 mg/kg BW did not result in significant improvements in any of the measures. Further, no other behavioral or physiological measures demonstrated significant effects of treatment. While cortisol is commonly used as an indicator of stress based on activation of the hypothalamic–pituitary-adrenocortical (HPA) axis, it should be noted that it can also be impacted by additional physiological factors, such as circadian rhythm and participation in vigorous activity (65). These external factors were controlled for as much as possible in the current study by keeping the timing of test sessions and blood sampling consistent for each dog throughout the study, and restricting activities such as walks and training sessions until after their post-test blood sample on test days.

It has also been suggested that cortisol increases in response to positive emotions of high arousal, such as excitement (66, 67), although a majority of research indicates a decrease of cortisol following positive emotions (reviewed by (68)). It is therefore possible that changes in cortisol may be due to changes in excitement caused by positive anticipation during the car journey. However, this is unlikely for the paradigm and population of dogs utilized in the current study. For these dogs, car travel is a novel experience and does not have any conditioned association with positive events, such as walks, which is the likely cause of excitement related to car travel in some pet dogs. Further, previous research using dogs from this population has demonstrated that they had elevated cortisol and were consistently rated as displaying negative valence emotions in response to car travel, and did not demonstrate an increase in cortisol in response to high arousal positive events, such as toy play and a treat chasing game (44). This supports the interpretation that the observed changes in cortisol in the current study are likely due to a reduction in stress.

Previous research conducted by the authors has demonstrated that CBD at 4 mg/kg BW resulted in a reduction in cortisol, whining, lip licking, and qualitative behavioral ratings using the same stress paradigm (5, 20). This effect was not replicated in the current study, despite a larger sample size being used, and implementing a cross-over design to reduce the impact of individual variation. One contributor to these different results may have been the different method of treatment delivery (capsule vs. treat), which could have potentially caused differences in dosing, bioavailability or absorption as discussed previously.

Further, the previous study may have had more dogs that absorbed and responded to CBD and/or responded with greater stress to the stress paradigm due to chance. Lastly, within the previous study it was observed that stress decreased over time in response to the car travel stress paradigm. Combined with the observed effect of study phase in the current study, it is possible that dogs habituated to the stress paradigm, leading to a lower stress response, and therefore less potential for CBD to have a stress reducing effect. However, visual inspection of the patterns in cortisol responses seen across the study population suggests the first test session resulted in the largest increase in cortisol, with moderate changes in cortisol observed for remaining test sessions with no further habituation. It should be noted that three dogs on the current study also participated in previous studies involving car travel and were therefore exposed to car travel for a total of nine times resulting in greater potential for habituation. However, these dogs showed a similar pattern in cortisol responses as the rest of the study population with a greater stress response to the first exposure. Alternatively, the previous study may represent a Type I error, where the significant relationship was identified due to chance rather than a true positive finding.

Within the current study, the presence of the blend appeared to have a beneficial effect on dog stress responses. While previous research using the same blend of L-Tryptophan and *α*-casozepine incorporated into a dry diet (Royal Canin CALM CANINE) has identified a stress-reducing effect, this was after 7 weeks of feeding (32). This is the first study to demonstrate a stress-reducing effect of L-Tryptophan and α-casozepine in dogs after a single dosage. Other studies using either L-tryptophan or α-casozepine in isolation have all been conducted using extended periods of daily feeding and had mixed results on their efficacy in reducing stress in dogs (30, 36, 42, 69). It is unknown whether the stress-reducing effect of the blend in the current study was due to a direct effect of the active ingredients on stress levels, or a synergistic effect when fed in combination with CBD. The current study did not analyze levels of tryptophan, α-casozepine, or their metabolites in the plasma and therefore the success of absorption and metabolism of the blend could not be established.

When measures of stress were analyzed against post-test CBD levels a significant reduction in HRV was identified suggesting that higher levels of CBD resulted in a lower stress response in the dogs. There was also a non-significant trend for increased standing behavior and decreased whining behavior as CBD levels increased. While these results did not meet the threshold for significance and therefore should be interpreted with caution, they may warrant further exploration.

Similarly, provision of the blend resulted in a non-significant trend for reduced change in cortisol from baseline to post-test, supporting further exploration of the potential stress-reducing effects of the blend independent of CBD. Comparison between indicators of stress and post-test CBD levels was conducted within the previous CBD study and did not demonstrate any significant effects (4). However, the previous study had a smaller sample size and was likely not sufficiently powered to detect differences in that analysis. Future studies should control for differences in CBD absorption either through dog selection, adjustment of dosages, or analysis against CBD plasma levels.

A number of physiological and behavioral measures did not demonstrate significant effects related to treatment. Individual differences in dog personality, previous experiences, and breed tendencies likely contributed to variation in behavioral and stress responses to the car travel stress paradigm, impacting the ability to detect significant effects. This may have been exacerbated by restrictions caused by the car environment. For example, large Labrador Retrievers did not have much space to move around in comparison to Norfolk Terriers. Conversely, Norfolk Terriers did not have much opportunity to see outside the vehicle as they were below the level of the window.

Variation was also introduced within the process of data capture. Video coders may have not reliably recorded or interpreted behavior. For example, some behaviors, such as QBA ratings showed only moderate reliability. While this was deemed sufficient for analysis, combined with the other sources of variation, it may have contributed to the lack of significant results for these measures. There may have also been error in data capture for heart rate based parameters caused by artifacts introduced by movement or device inaccuracy. The current study utilized the Polar Verity Sense device mounted to a collar over an area of the neck where the hair was shaved. This specific device has not been validated for use in dogs, but similar devices using photoplethysmography (PPG) to measure heart rate have been demonstrated to be accurate if hair is shaved (70, 71). While the data in the current study fell within expected ranges for each breed, it is possible that variation was introduced if the collar shifted away from the shaved area, or if dogs were more active.

Finally, variation introduced by differences in absorption and response to CBD likely contributed to not being able to detect significant differences in these additional behavioral and physiological measures. It should also be noted that the study was powered to detect differences in the primary measure of cortisol, and therefore the study may have been underpowered to detect significant differences in the secondary measures, especially with these added contributors of variation.

## Conclusion

The results obtained from the current study suggest that a semi-moist treat containing a dose of 2 mg/kg BW CBD in combination with L-Tryptophan and *α*-casozepine resulted in a mild stress-reducing effect in dogs in response to car travel compared to a placebo. However, previous findings of efficacy of CBD when provided at a dose of 4 mg/kg BW were not replicated. This failure to replicate findings was likely due to individual variation in dogs’ stress responses to the car travel stress paradigm, as well as individual differences in CBD absorption. It is recommended that additional studies are conducted to further explore factors contributing to differences in CBD absorption and metabolism, as well as examine potential synergistic effects between CBD, L-Tryptophan and α-casozepine.

## Data Availability

The raw data supporting the conclusions of this article will be made available by the authors, without undue reservation.
