# Supplementary material for: Treats containing cannabidiol, L-tryptophan and α-casozepine have a mild stress-reducing effect in dogs
Source: Front Vet Sci. 2025 Jul 29;12:1632868. doi: 10.3389/fvets.2025.1632868 (PMC12339541; doi:10.3389/fvets.2025.1632868)
Supplement: Supplementary file 1 [file Table_1.DOCX]

**Supplementary Material**

Table S1 – Demographic information for dogs that participated in the study, including breed (BE: Beagle, LR: Labrador Retriever, NT: Norfolk Terrier), age at the start of the trial (years), sex (M: male, F: female), neuter status (N: neutered/spayed. E: entire), body weight (kg) at the first weighing timepoint, and latin square group defining treatment order.

| Animal ID | Breed | Age | Sex | Neuter Status | Body Weight | Latin Square Group |
| --- | --- | --- | --- | --- | --- | --- |
| BE07076 | BE | 4.5 | M | N | 16.6 | 4 |
| BE07248 | BE | 4.0 | F | N | 11.3 | 3 |
| BE07516 | BE | 3.5 | M | N | 13.9 | 1 |
| BE07780 | BE | 2.0 | M | N | 13.2 | 4 |
| BE07807 | BE | 1.9 | F | N | 11.1 | 1 |
| BE07815 | BE | 1.6 | M | N | 15.5 | 1 |
| BE07816 | BE | 1.6 | M | N | 13.8 | 1 |
| BE07820 | BE | 1.6 | M | N | 16.5 | 2 |
| BE07827 | BE | 1.5 | F | E | 10.3 | 4 |
| LR06955 | LR | 5.1 | M | N | 34.3 | 3 |
| LR06957 | LR | 5.1 | M | N | 34.0 | 2 |
| LR07143 | LR | 4.3 | M | N | 27.9 | 3 |
| LR07196 | LR | 4.1 | F | N | 26.5 | 2 |
| LR07197 | LR | 4.1 | F | N | 25.6 | 1 |
| LR07198 | LR | 4.1 | F | N | 22.1 | 3 |
| LR07200 | LR | 4.1 | M | N | 29.1 | 4 |
| LR07371 | LR | 3.7 | M | N | 24.6 | 4 |
| LR07375 | LR | 3.7 | M | N | 32.6 | 2 |
| LR07376 | LR | 3.7 | F | N | 24.6 | 4 |
| LR07378 | LR | 3.7 | F | N | 26.8 | 1 |
| LR07609 | LR | 3.2 | F | N | 21.6 | 4 |
| LR07635 | LR | 2.8 | M | N | 33.3 | 4 |
| LR07636 | LR | 2.8 | M | N | 30.5 | 2 |
| LR07640 | LR | 2.6 | M | N | 27.4 | 4 |
| LR07641 | LR | 2.6 | M | N | 30.8 | 2 |
| LR07645 | LR | 2.6 | F | N | 27.5 | 2 |
| LR07647 | LR | 2.7 | M | N | 29.3 | 1 |
| LR07648 | LR | 2.7 | M | N | 31.0 | 3 |
| LR07718 | LR | 2.3 | M | N | 29.0 | 4 |
| LR07719 | LR | 2.4 | M | N | 29.0 | 4 |
| LR07720 | LR | 2.3 | M | N | 32.7 | 3 |
| LR07787 | LR | 1.9 | M | N | 27.8 | 1 |
| LR07788 | LR | 1.9 | F | E | 28.5 | 1 |
| LR07790 | LR | 1.9 | F | N | 25.3 | 3 |
| LR07791 | LR | 1.9 | F | N | 22.8 | 3 |
| NT06439 | NT | 6.7 | M | N | 7.9 | 2 |
| NT06441 | NT | 6.7 | M | N | 4.6 | 2 |
| NT06573 | NT | 6.5 | M | N | 5.4 | 3 |
| NT06596 | NT | 6.2 | F | N | 3.7 | 3 |
| NT06640 | NT | 5.9 | M | N | 7.0 | 4 |
| NT06641 | NT | 5.9 | F | N | 5.2 | 4 |
| NT06642 | NT | 5.9 | F | N | 5.4 | 3 |
| NT06974 | NT | 5.0 | F | N | 4.9 | 2 |
| NT06976 | NT | 5.0 | F | N | 4.9 | 1 |
| NT07000 | NT | 4.8 | F | N | 4.0 | 3 |
| NT07001 | NT | 4.8 | M | N | 4.7 | 1 |
| NT07004 | NT | 4.8 | M | N | 5.4 | 2 |
| NT07181 | NT | 4.2 | M | N | 6.4 | 3 |
| NT07367 | NT | 3.7 | M | N | 5.6 | 2 |
| NT07561 | NT | 3.3 | F | N | 4.9 | 4 |
| NT07602 | NT | 3.1 | F | N | 5.6 | 4 |
| NT07627 | NT | 2.9 | F | E | 4.7 | 1 |
| NT07697 | NT | 2.6 | M | N | 5.9 | 2 |
| NT07714 | NT | 2.2 | F | N | 5.1 | 2 |
